# Phenotypes of Atopic Dermatitis and Development of Allergic Diseases

**DOI:** 10.1001/jamanetworkopen.2025.15094

**Published:** 2025-06-12

**Authors:** Alexandra R. Sitarik, Amy A. Eapen, Jocelyn M. Biagini, Daniel J. Jackson, Christine L. M. Joseph, Haejin Kim, Lisa J. Martin, Katherine Rivera-Spoljaric, Eric M. Schauberger, Ganesa Wegienka, Casper Bendixsen, Agustín Calatroni, Soma Datta, Diane R. Gold, Lisa Gress, Tina V. Hartert, Christine C. Johnson, Gurjit K. Khurana Hershey, Fernando D. Martinez, Rachel L. Miller, Christine M. Seroogy, Sweta Singh, Anne L. Wright, James E. Gern, Anne Marie Singh

**Affiliations:** 1 Department of Public Health Sciences, Henry Ford Health, Detroit, Michigan; 2 Department of Epidemiology and Biostatistics, College of Human Medicine, Michigan State University, East Lansing; 3 Division of Allergy and Clinical Immunology, Department of Medicine, Henry Ford Health, Detroit, Michigan; 4 Division of Asthma Research, Cincinnati Children’s Hospital Medical Center, Cincinnati, Ohio; 5 Department of Pediatrics, University of Cincinnati College of Medicine, Cincinnati, Ohio; 6 Department of Pediatrics, Division of Allergy, Immunology, and Rheumatology, University of Wisconsin School of Medicine and Public Health, Madison; 7 Department of Pediatrics, Cincinnati Children’s Hospital Medical Center, Cincinnati, Ohio; 8 Department of Pediatrics, Washington University School of Medicine, St Louis, Missouri; 9 Department of Obstetrics, Gynecology and Reproductive Biology, College of Human Medicine, Michigan State University, East Lansing; 10 National Farm Medicine Center, Marshfield Clinic Research Institute, Marshfield, Wisconsin; 11 Rho Inc, Federal Research Operations, Durham, North Carolina; 12 Channing Division of Network Medicine, Brigham and Women’s Hospital and Harvard Medical School, Boston, Massachusetts; 13 Department of Environmental Health, Harvard T. H. Chan School of Public Health, Boston, Massachusetts; 14 Department of Medicine, Division of Allergy, Pulmonary, and Critical Care Medicine, Vanderbilt University School of Medicine, Nashville, Tennessee; 15 Department of Pediatrics, Vanderbilt University School of Medicine, Nashville, Tennessee; 16 Asthma and Airways Disease Research Center, University of Arizona, Tucson; 17 Division of Pulmonary and Sleep Medicine, Department of Pediatrics, College of Medicine, University of Arizona, Tucson; 18 Division of Clinical Immunology, Icahn School of Medicine at Mount Sinai, New York, New York; 19 Clinical and Health Informatics Institute, School of Medicine and Public Health, University of Wisconsin, Madison

## Abstract

**Importance:**

Atopic dermatitis (AD) is the most common inflammatory disease in childhood, and children with AD are more likely to develop other allergic diseases, including food allergy, allergic rhinitis, and asthma.

**Objective:**

To determine the phenotypes of AD expression across 12 US birth cohorts and identify factors associated with phenotype and development of allergic diseases.

**Design, Setting, and Participants:**

This cohort study compiled longitudinal data from 12 observational US birth cohorts across decades (children born from April 1980 to June 2019) in the Environmental Influences on Child Health Outcomes (ECHO) Children’s Respiratory and Environmental Workgroup with follow-up to September 2022. Participants were enrolled prenatally; children with 3 or more AD assessments across the first 84 months of life were included in analyses. Data were analyzed from December 2020 to April 2024.

**Exposures:**

Exposures included decade of birth, cohort type (population-based or high-risk), family history of asthma (mother, father, or sibling), birth order, gestational age at birth, delivery mode, breastfeeding, pet exposure, antibiotic use, environmental tobacco smoke exposure, allergic sensitization, peripheral blood eosinophil count, and total IgE.

**Main Outcomes and Measures:**

Primary outcomes were AD phenotype, food allergy, allergic rhinitis, asthma, and wheeze. Longitudinal latent class analysis was used to identify underlying longitudinal patterns of AD expression, and associations of AD phenotype with allergic outcomes were examined using logistic regression, multinomial logistic regression, and linear regression.

**Results:**

In 5314 children from 9 cohorts (1896 born in the 2000s [35.7%]; 2585 female [48.6%]; 1083 Black or African American [20.4%]; 3344 White [62.9%]; 350 other reported race [6.6%; including 8 American Indian or Alaska Native (0.2%); 58 Asian (1.1%); 4 Native Hawaiian or Pacific Islander (0.1%) and 280 multiracial or with any race not otherwise specified (5.3%)]), 3382 (63.6%) were from a population-based cohort, while 1932 (36.4%) were from a high-risk cohort. AD prevalence ranged from 24.1% (540 children) to 28.4% (1156 children) at each time point, and 5 phenotypes of AD were identified: transient early AD, early AD with potential reoccurrence, late-onset AD, persistent AD, and minimal or no AD. Compared with White children, Black children were at higher risk for AD (transient early AD: aOR, 3.26; 95% CI, 2.06-5.18; early AD with potential reoccurrence: aOR, 3.72; 95% CI, 2.35-5.90; persistent AD: aOR, 2.01; 95% CI, 1.54-2.63), as were children with other reported race (transient early AD: aOR, 2.31; 95% CI, 1.13-4.70; early AD with potential reoccurrence: aOR, 3.27; 95% CI, 1.73-6.18). Female children were significantly less likely to have early AD with potential reoccurrence (aOR, 0.45; 95% CI, 0.27-0.74) and persistent AD (aOR, 0.60; 95% CI, 0.49-0.74) than male children. Compared with miniml or no AD, phenotypes with early AD expression were associated with food allergy (transient early AD: adjusted odds ratio [aOR], 2.15; 95% CI, 1.48-3.08; early AD with potential reoccurrence: aOR, 2.43; 95% CI, 1.66-3.50; persistent AD: aOR, 2.26; 95% CI, 1.84-2.78), later AD expression was associated with allergic rhinitis (late-onset AD: aOR, 1.84; 95% CI, 1.38-2.43; persistent AD: aOR, 2.02; 95% CI, 1.64-2.48), and any AD disease was associated with asthma.

**Conclusions and Relevance:**

In this birth cohort study of 5314 children, timing of AD expression was associated with increased risk for atopic march pathways. Identifying risk factors for AD phenotypes may inform targeted therapeutic prevention strategies.

## Key Points

**Question:**

Is the phenotypic expression of atopic dermatitis (AD) associated with the development of other allergic diseases, and what factors are associated with each phenotype?

**Findings:**

This cohort study of 5314 children from 12 US birth cohorts found that AD in children was common and identified 5 distinct AD phenotypes with different associations with comorbidities. Phenotypes with early AD expression were associated with food allergy, phenotypes with later AD expression with allergic rhinitis, and any AD phenotype with asthma.

**Meaning:**

In this cohort study, timing of AD expression was associated with development of other allergic diseases, suggesting that the timing of skin barrier disruption plays an important role in atopic march pathways.

## Teaser

This cohort study of children from 12 US birth cohorts characterizes phenotypes of atopic dermatitis expression and identifies factors associated with each phenotype and development of allergic diseases.

## Introduction

Atopic dermatitis (AD) is a chronic inflammatory skin disease characterized by intense pruritus and eczematous lesions that substantially impair quality of life.^1,2,3^ The pathogenesis of AD includes genetic factors and environmental exposures, and impaired epidermal barrier is a key feature leading to inflammatory responses and AD.^4,5,6,7^ AD is most common in childhood, impacting 20% of children globally.^8^ Approximately 60% of those with AD develop it in the first year of life,^9^ and incidence has increased over the last few decades,^8^ particularly in nonendemic countries.^10^

Although AD is most commonly seen in young children, the onset and resolution of the disease also varies widely. AD phenotypes have been identified based on the natural history of the disease and associations with other allergic diseases.^11,12^ However, how AD phenotypes vary across diverse populations is not well understood.

AD is associated with the development of comorbidities such as food allergy, allergic rhinitis, and asthma.^9,13,14,15,16,17^ For example, approximately 20% of children with mild AD and 60% of children with severe AD go on to develop asthma.^18^ Longitudinal observational studies^19,20,21^ indicate that some children have an atopic march, referring to the progression from atopic dermatitis to food allergy, allergic rhinitis, and asthma. However, only a subset of children with AD develop these comorbidities.

We aim to understand and characterize the different AD phenotypic patterns among children participating in the Children’s Respiratory and Environmental Workgroup (CREW), a consortium of 12 US birth cohorts.^22^ The study goals were to identify personal characteristics and early life exposures associated with the development of disease and examine risk across AD phenotypes for developing other allergic diseases.

## Methods

This cohort study followed the Strengthening the Reporting of Observational Studies in Epidemiology (STROBE) reporting guideline. Study participants were drawn from CREW, which was funded by the National Institutes of Health Environmental Influences on Child Health Outcomes (ECHO) program.^23,24^ The investigators of the 12 participating US birth cohorts developed a data sharing protocol and data use agreements that were approved by the local institutional review boards for each participating cohort.^22^ Written informed consent or parent or guardian permission was obtained along with child assent as appropriate for participation in specific cohorts and the CREW protocol. Eligibility criteria, sources and methods of participant selection have been reported.^22^

Definitions for each variable in each cohort were compiled, and harmonized variable definitions that permitted consistent ascertainment across all of the cohorts were developed. The pooled dataset included information collected from children born between January 1980 and June 2019, with follow-up to September 2022.

### Clinical Outcomes

#### AD

AD was determined by report of current physician diagnosis in the past year (Columbia Center for Children’s Environmental Health, Tucson Children’s Respiratory Study [TCRS], Infant Immune Study, Childhood Origins of Asthma [COAST], Urban Environment and Childhood Asthma [URECA], and Epidemiology of Home Allergens and Asthma Study [EHAAS] cohorts), validated International Study of Asthma and Allergies in Childhood (ISAAC) question responses (Wisconsin Infant Study Cohort [WISC]), parental report of AD (Childhood Allergy Study [CAS] and Respiratory Syncytial Virus Infection During Infancy and Asthma During Childhood [INSPIRE] cohorts), or by global assessment of the cohort allergist based on history and physical examination at a study visit (Cincinnati Childhood Allergy and Air Pollution Study [CCAAPS]; Microbes, Allergy, Asthma Precision Prevention [MAAP]; and Wayne County Health, Environment, Allergy, and Asthma Longitudinal Study [WHEALS] cohorts). For this study, we determined AD at most once per year. If AD was collected at multiple time points within a year, yes at any time in the previous year was considered yes for that year. AD was determined at each year until 84 months of age in each cohort. Because we sought to determine the expression of AD over time, cohorts with fewer than 3 time points assessed were not included (MAAP, TCRS, and WHEALS cohorts).

#### Food Allergy

Food allergy was defined as a physician-reported diagnosis of food allergy (MAAP, URECA, INSPIRE, EHAAS, and CAS cohorts), parental report of food allergy or symptoms consistent with food allergy (WISC and COAST cohorts), or an investigator global assessment for food allergy (CCAAPS and WHEALS cohorts). The latter definition included a clinical diagnosis of food allergy either at the time of a study visit by a study clinician or by a clinician who reviewed food-induced symptoms, skin testing, and specific IgE to foods.

#### Asthma

Asthma was defined as ever having a physician diagnosis of asthma, as reported by the parent (usually the mother).^25^ Similarly, sibling or parental asthma were defined as parental report of diagnosis of asthma at any time.

#### Allergic Rhinitis

Allergic rhinitis was defined as a physician diagnosis of allergic rhinitis or hay fever by parental report at annual study visits. The definition was assessed in time windows of 2 to 4 years and 5 to 7 years of age.

#### Early Wheeze

These variables were previously harmonized.^25^ Briefly, all cohorts asked questions about whether a participant wheezed during a specified period of time, usually over the past 12 months. Binary (yes or no) variables indicated if that participant wheezed in each year from birth to age 5 years. The remaining definitions are included in the eMethods in Supplement 2.

### Statistical Analysis

Statistical significance was prespecified at a 2-sided *P* < .05 throughout analyses. All analyses were performed using R version 4.2.3 (R Project for Statistical Computing) or Mplus version 8.9 (Muthén & Muthén) from December 2020 (month first data request fulfilled) to November 2024 (month of steering committee approval).

Longitudinal latent class analysis was used to identify underlying longitudinal patterns of AD expression,^26,27^ using indicators for AD at each time point as manifest variables. This approach was selected over growth mixture modeling and latent class growth analysis to allow for more flexibility in model parameters by not imposing any assumptions about the function form of the change process.^28^ Because the goal was to assess AD over time, CREW children were retained for analysis if they had nonmissing AD data at 3 or more time points. This excluded children from the MAAP, WHEALS, and TCRS cohorts, and 17% (range, 2%-42%) of children from the remaining 9 cohorts. Distribution of AD assessments was similar throughout the included cohorts. (eFigure 1 in Supplement 2). Model selection is discussed in more detail in the eMethods in Supplement 2.

The association of AD phenotype with allergic outcomes was examined using logistic regression for binary outcomes, multinomial logistic regression for outcomes with more than 2 categories, and linear regression for continuous outcomes. To account for uncertainty in class assignment, the maximum posterior probability for AD phenotype assignment was used as a subject weight. Models were again adjusted for cohort type, decade of birth, child sex, and child race. Race categories included American Indian or Alaska Native, Asian, Black or African American, Native Hawaiian or Pacific Islander, White, other race or multiracial (ie, any race not listed), and missing race. In analyses, American Indian or Alaska Native, Asian, and Native Hawaiian or Pacific Islander were collapsed into the other or multiracial category due to small sample sizes. Race was included to assess associations given known racial disparities in incidence, prevalence, and severity of atopic dermatitis.

## Results

**Figure 1.**
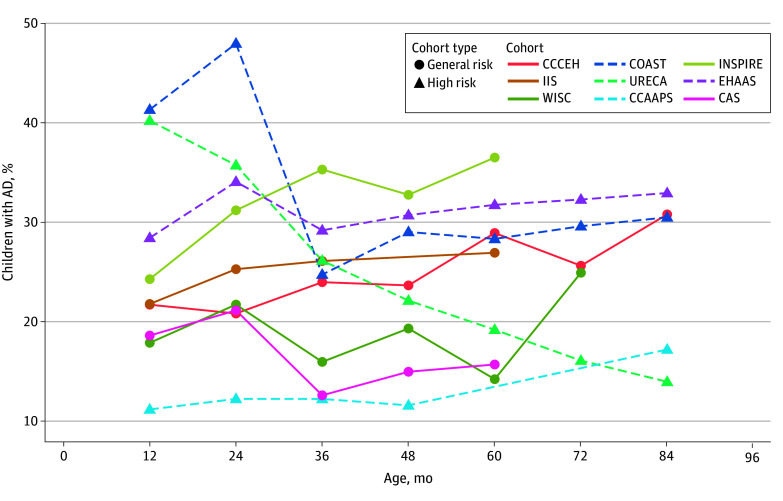
Prevalence of Atopic Dermatitis (AD) by Age and Cohort Each individual cohort is represented by a distinct color. General-risk (or population-based) cohorts are represented with circles and solid lines, and high-risk cohorts are represented with triangles and dotted lines. CAS indicates Childhood Allergy Study; CCAAPS, Cincinnati Childhood Allergy and Air Pollution Study; CCCEH, Columbia Center for Children’s Environmental Health; COAST, Childhood Origins of Asthma; EHAAS, Epidemiology of Home Allergens and Asthma Study; IIS, Infant Immune Study; INSPIRE, Respiratory Syncytial Virus Infection During Infancy and Asthma During Childhood in the USA; URECA, Urban Environment and Childhood Asthma; WISC, Wisconsin Infant Study Cohort.

The study population consisted of 5314 children from 9 birth cohorts, with 3382 children (63.6%) from a population-based cohort and 1932 children (36.4%) from a high-risk cohort, selected based on family history of allergic disease (Table 1). The children were recruited across multiple decades (from the 1980s to 2010s; 1896 born in the 2000s [35.7%]). Of all participants, 2729 were male (51.4%) and 2585 (48.6%) were female. The majority of participants were Black or African American (1083 children [20.4%]), White (3344 children [62.9%]), or reported other race (350 [6.6%]; including 8 American Indian or Alaska Native [0.2%]; 58 Asian [1.1%]; 4 Native Hawaiian or Pacific Islander [0.1%] and 280 multiracial or with any race not otherwise specified [5.3%)]) by parental report. Overall, AD was common among participants, with a prevalence between 24.1% (540 children) and 28.4% (1156 children) combined across cohorts at each year. AD prevalence varied across cohorts (Figure 1). High-risk cohorts had significantly higher prevalence of AD at 12 months, but significantly lower prevalence from 36 to 60 months, as well as at 84 months (eFigure 2 in Supplement 2). Approximately one-quarter of the participants reported having a physician diagnosis of AD in the first year of life (1241 participants [23.4%]) (eTable 1 in Supplement 2). Other information on participant characteristics can be found in eTable 1 in Supplement 2.

**Table 1.** Characteristics of Participants in the Children’s Respiratory and Environmental Workgroup Included in the Analysis Subset

Characteristic	Participants, No. (%) (N = 5314)
Cohort	
Childhood Allergy Study	663 (12.5)
Cincinnati Childhood Allergy and Air Pollution Study	654 (12.3)
Columbia Center for Children’s Environmental Health	586 (11.0)
Childhood Origins of Asthma	276 (5.2)
Epidemiology of Home Allergens and Asthma Study	484 (9.1)
Infant Immune Study	373 (7.0)
Respiratory Syncytial Virus Infection During Infancy and Asthma During Childhood	1604 (30.2)
Microbes, Allergy, Asthma Precision Prevention^a^	0
Tucson Children’s Respiratory Study^a^	0
Urban Environment and Childhood Asthma	518 (9.7)
Wayne County Health, Environment, Allergy, and Asthma Longitudinal Study^a^	0
Wisconsin Infant Study Cohort	156 (2.9)
Cohort type^b^	
General Risk	3382 (63.6)
High Risk	1932 (36.4)
Decade of birth	
1980s	663 (12.5)
1990s	995 (18.7)
2000s	1896 (35.7)
2010s	1756 (33.0)
Missing	4 (0.1)
Child sex	
Male	2729 (51.4)
Female	2585 (48.6)
Child race	
American Indian or Alaska Native^c^	8 (0.2)
Asian^c^	58 (1.1)
Black or African American	1083 (20.4)
Native Hawaiian or Pacific Islander^c^	4 (0.1)
White	3344 (62.9)
Other or multiracial^d^	280 (5.3)
Missing	537 (10.1)

^a^ All children from these cohorts were excluded from analyses because fewer than 3 atopic dermatitis time points were able to be harmonized.

^b^ General-risk cohorts were population based, while high-risk cohorts were enriched for a family history of atopy.

^c^ These categories were collapsed into the other or multiracial category for analysis due to small sample sizes.

^d^ Other or multiracial comprises participants who self-reported as other (ie, a race not listed) or as multiracial.

**Figure 2.**
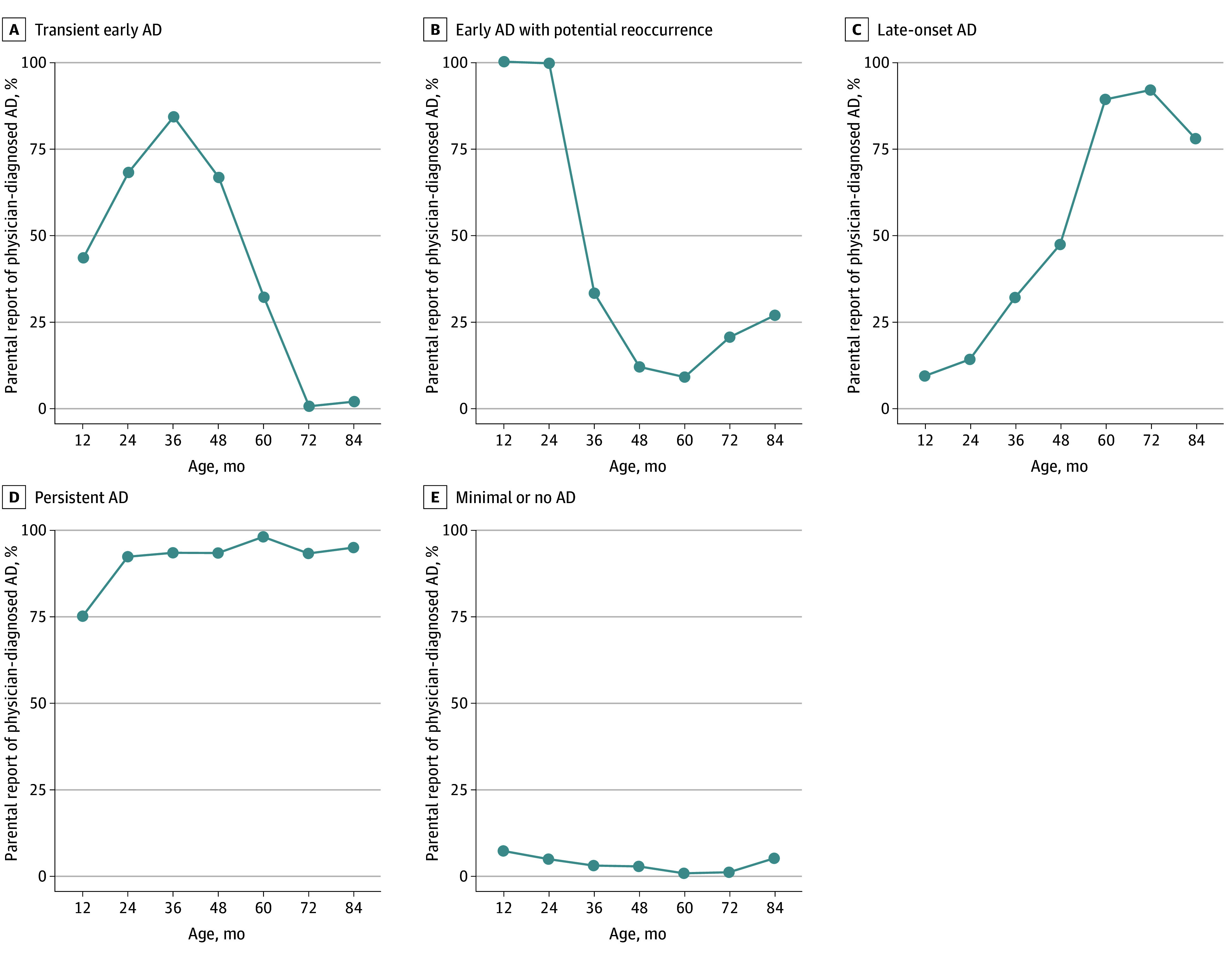
Description of the 5 Atopic Dermatitis (AD) Phenotypes Identified in the Children’s Respiratory and Environmental Workgroup Summary of AD expression across time for each phenotype identified is shown.

A 5-class longitudinal latent class analysis model was selected based on fit statistics (eTable 2 in Supplement 2) and clinical interpretability. The classes were transient early AD, which had an increasing prevalence of AD that peaked at age 3 years before declining (261 participants [4.9%]); early AD with potential reoccurrence, which demonstrated a high prevalence of AD in the first 2 years, followed by reduced prevalence and a rebound by age 6 to 7 years (229 participants [4.3%]); late-onset AD, in which there was little AD in the first 2 years of life, and the prevalence of AD gradually increased over time and peaked at approximately 6 years (478 participants [9.0%]); persistent AD, which demonstrated a high prevalence of AD throughout childhood (821 participants [15.4%]); and minimal or no AD (3525 participants [66.3%]), which had a low prevalence of AD throughout childhood (Figure 2). The entropy of the 5-class model was relatively high at 0.79, demonstrating good classification accuracy. The median (IQR) maximum posterior probability within each class ranged from 0.64 (0.56-0.90) for late-onset AD to 0.98 (0.95-0.98) for minimal or no AD (eFigure 3 in Supplement 2). Each of the 9 cohorts were generally well-represented across classes, with no single class composed entirely by a particular cohort (eTable 3 in Supplement 2).

### Factors Associated With Increased Risk for AD Phenotypes

We evaluated personal and environmental risk factors for AD phenotypes after adjusting for cohort type, decade of birth, sex of child, and child race. Children recruited from high-risk cohorts were more likely to have early AD with potential reoccurrence (adjusted odds ratio [aOR], 1.79; 95% CI, 1.05-3.06), but less likely to have late (aOR, 0.53; 95% CI, 0.32-0.88) or persistent (aOR, 0.38; 95% CI, 0.17-0.84) AD^29^ (Table 2). Decade of birth was not associated with AD phenotype after adjustment (Table 2).

**Table 2.** Associations of Household, Family, and Child Characteristics With AD phenotype (N = 5314)

Characteristic	Participants, No.	Risk of AD by phenotype, adjusted OR (95% CI)^a^			
		Transient early AD (n = 261 [4.9%])	Early AD with potential reoccurrence (n = 229 [4.3%])	Late-onset AD (n = 478 [9.0%])	Persistent AD (n = 821 [15.4%])
Personal risk factors					
Cohort type: high vs general risk	4777	2.79 (0.80-9.80)	1.79 (1.05-3.06)^b^	0.53 (0.32-0.88)^c^	0.38 (0.17-0.84)^c^
Decade of birth: 2000s-2010s vs 1980s-1990s	4777	0.42 (0.12-1.48)	0.72 (0.43-1.21)	1.15 (0.70-1.88)	1.36 (0.69-2.68)
Child sex: female vs male	4777	1.21 (0.80-1.83)	0.45 (0.27-0.74)^c^	1.17 (0.85-1.60)	0.60 (0.49-0.74)^c^
Child race: Black or African American compared with White	4427	3.26 (2.06-5.18)^b^	3.72 (2.35-5.90)^b^	1.19 (0.78-1.82)	2.01 (1.54-2.63)^b^
Child race: other or multiracial compared with White^d^	3694	2.31 (1.13-4.70)^b^	3.27 (1.73-6.18)^b^	0.80 (0.39-1.65)	1.11 (0.72-1.71)
Maternal asthma: yes vs no	3040	1.08 (0.63-1.84)	2.66 (1.55-4.57)^b^	1.13 (0.65-1.97)	2.08 (1.46-2.95)^b^
Paternal asthma: yes vs no	3057	1.76 (1.04-2.99)^b^	1.23 (0.70-2.18)	1.33 (0.73-2.43)	1.59 (1.11-2.28)^b^
Sibling asthma: yes vs no	2753	1.31 (0.73-2.35)	1.45 (0.75-2.79)	1.55 (0.90-2.66)	1.52 (1.10-2.09)^b^
First born child: yes vs no	1892	0.76 (0.23-2.51)	0.98 (0.46-2.10)	1.19 (0.67-2.10)	0.87 (0.63-1.20)
Gestational age per 1-week increase	4691	0.79 (0.69-0.91)^c^	1.21 (0.97-1.50)	1.05 (0.92-1.21)	1.04 (0.93-1.16)
Modifiable risk factors					
Delivery mode: cesarean vs vaginal	3083	1.15 (0.69-1.92)	1.15 (0.66-2.01)	1.05 (0.68-1.62)	1.04 (0.82-1.32)
Ever breastfed: yes vs no	4184	0.90 (0.56-1.45)	0.88 (0.50-1.54)	1.17 (0.75-1.83)	1.60 (1.22-2.11)^b^
Antibiotic use in the first year of life: yes vs no	3006	1.07 (0.64-1.77)	1.07 (0.59-1.93)	1.21 (0.79-1.83)	1.30 (1.01-1.67)^b^
Daycare in the first year of life: yes vs no	3613	1.08 (0.64-1.80)	1.03 (0.64-1.66)	1.47 (0.99-2.19)	1.33 (1.03-1.71)^b^
Dogs in the first year of life: yes vs no	4729	1.15 (0.64-2.08)	0.58 (0.30-1.13)	0.73 (0.51-1.05)	0.76 (0.61-0.96)^c^
Cats in the first year of life: yes vs no	4728	0.88 (0.54-1.45)	1.32 (0.80-2.15)	0.49 (0.31-0.78)^c^	0.85 (0.68-1.06)
Maternal smoking during first year of life: yes vs no	4300	0.97 (0.58-1.62)	0.86 (0.51-1.46)	0.69 (0.42-1.15)	0.58 (0.42-0.80)^c^
Other household smokers during first year of life: yes vs no	4538	1.31 (0.76-2.26)	0.92 (0.56-1.49)	1.00 (0.71-1.43)	0.82 (0.64-1.04)

Abbreviations: AD, atopic dermatitis; OR, odds ratio.

^a^ ORs and 95% CIs were estimated using the multinomial logistic regression for each covariate with the 3-step procedure described by Asparouhov et al.^29^ All ORs are relative to the minimal or no AD class, which was included 3525 children (66.3%), and are adjusted for cohort type, decade of birth, child sex, and child race.

^b^ Significant ORs greater than 1.

^c^ Significant ORs less than 1.

^d^ Other included American Indian or Alaska Native, Asian, Native Hawaiian or Pacific Islander, and any race not otherwise specified.

Several child characteristics differed among the phenotypes. Female children were significantly less likely to have early AD with potential reoccurrence (aOR, 0.45; 95% CI, 0.27-0.74) and persistent AD (aOR, 0.60; 95% CI, 0.49-0.74) than male children (Table 2). Black or African American children were more likely to have transient early AD (aOR, 3.26; 95% CI, 2.06-5.18), early AD with potential reoccurrence (aOR, 3.72; 95% CI, 2.35-5.90), and persistent AD (aOR, 2.01; 95% CI, 1.54-2.63) compared with White children. Multiracial children and children with other race reported were more likely to have transient early AD (aOR, 2.31; 95% CI, 1.13-4.70) and early AD with potential reoccurrence (aOR, 3.27; 95% CI, 1.73-6.18) compared with White children.

Parental asthma had the largest-magnitude association with early AD phenotypes, with children of asthmatic mothers being significantly more likely to have early AD with potential reoccurrence (aOR, 2.66; 95% CI, 1.55-4.57), and children of asthmatic fathers being significantly more likely to have transient early AD (aOR, 1.76; 95% CI, 1.04-2.99) (Table 2). In addition, maternal, paternal, and sibling asthma were all significantly associated with the risk of persistent AD. Older gestational age at birth was associated with lower odds of transient early AD (aOR, 0.79; 95% CI, 0.69-0.91 per 1-week increase). Breastfeeding, antibiotic use, and daycare in the first year of life were all associated with increased odds of persistent AD, while dog and cat exposure in the first year of life was associated with protection against AD development (Table 2 and eTable 4 in Supplement 2).

We used conditional inference trees to identify single and grouped variables associated with AD phenotype. The model first split on child race, then cohort type, decade of birth, parental history of asthma, and breastfeeding, which resulted in 9 terminal nodes (eFigure 4 in Supplement 2). Node 8—which was enriched for Black children, multiracial children, or children with other reported race from high-risk cohorts born after the 1990s—had the highest probability of transient early AD (68 of 626 children [10.9%]) and early AD with potential reoccurrence (75 of 626 children [12.0%]). Node 14—which included White children born before the 2000s who were not breastfed and had a maternal (but not a paternal) history of asthma—had the highest probability of late-onset AD (13 of 93 children [14.0%]). Node 5—which included Black children, multiracial children, and children with other reported race from general risk cohorts born after the 2000s—had the highest probability of persistent AD (136 of 445 children [30.6%]). Lastly, Node 12—which included White children born before the 2000s with no parental history of asthma—had the highest probability of minimal or no AD (1195 of 1562 children [76.5%]).

### Associations With Comorbidities and Th2 Biomarkers

**Figure 3.**
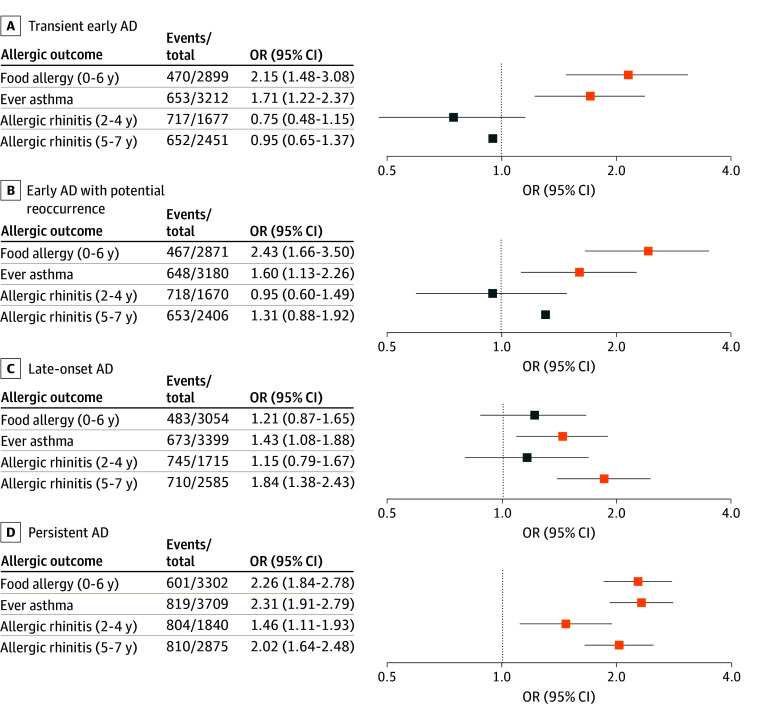
Atopic Dermatitis (AD) Phenotype and Allergic Disease Outcomes Odds ratios (ORs) are adjusted for cohort type, decade of birth, child sex, and child race. Orange squares represent AD phenotypes with significantly increased odds (relative to the minimal or no AD phenotype) for the allergic disease shown. Blue squares represent nonsignificant ORs. Columns show the total number of events relative to the total number of children for each allergic outcome and comparison (each AD phenotype vs minimal or no AD), as well as ORs and 95% CIs.

Using minimal or no AD as the reference group, phenotypes with early expression of AD and persistent AD were significantly associated with higher odds of food allergy in the first 6 years of life (transient early AD: aOR, 2.15; 95% CI, 1.48-3.08; early AD with potential reoccurrence: aOR, 2.43; 95% CI, 1.66-3.50; persistent AD: aOR, 2.26; 95% CI, 1.84-2.78). Late-onset (aOR, 1.84; 95% CI, 1.38-2.43) and persistent AD (aOR, 2.02; 95% CI, 1.64-2.48) were associated with higher odds of allergic rhinitis from 5 to 7 years of age (Figure 3). All 4 AD phenotypes were significantly associated with asthma, but the largest effect size was observed for persistent AD (aOR, 2.31; 95% CI, 1.91-2.79). Children with persistent AD were also more likely to have persistent wheeze through age 1 to 5 years, whereas children with transient early AD were more likely to have wheeze only between 2 and 4 years.

Additionally, we assessed the association of T2 biomarkers with AD phenotypes, using the minimal or no phenotype as the reference group (eTable 5 in Supplement 2). Sensitization to foods, which typically occurs during the preschool years, was associated with transient early and persistent AD phenotypes (eTable 5 in Supplement 2). Egg and peanut sensitization had a larger-magnitude association with AD phenotype compared with milk. In contrast, perennial sensitization was more common in children with early AD with potential reoccurrence and persistent AD. Persistent AD was also significantly associated with seasonal aeroallergen sensitization.

Children with persistent AD had increased odds of having medium (aOR, 2.09; 95% CI, 1.56-2.81]) to high (aOR, 3.71; 95% CI, 2.52-5.45) levels of total IgE throughout childhood (eTable 5 in Supplement 2). Further, compared with children with minimal or no AD, childhood eosinophil counts were 43.9% (95% CI, 2.2%-102.6%) higher for children with transient early AD and 62.9% (95% CI, 23.9%-114.1%) higher for children with persistent AD.

## Discussion

Using longitudinal data from a diverse group of children participating in CREW-ECHO, this cohort study found that approximately one-third of children had AD over the first 7 years of life. Analysis of AD across time identified 5 phenotype groups associated with the presence, onset, and persistence of AD. The prevalence of specific phenotypes was associated with sex, parent-reported race, and early life exposures such as antibiotics and pets in the home. Notably, the phenotypes had distinct associations with food allergy, allergic rhinitis, and asthma. Our results highlight that early life AD patterns have distinct risk factors and associations with atopic comorbidities.

While one-third of our participants had a physician diagnosis of AD over the first 7 years of life, when considering each time point separately, physician-diagnosed AD ranged from 7.9% to 25.3%, with earlier time points having higher rates of AD. The overall rate of AD was higher than reported in some prior studies,^30,31^ and interestingly, overall rates did not statistically differ between population-based and high-risk cohorts. Given the variability in AD onset and persistence, examining a single time point as in prior studies may underestimate the cumulative risk. It is also possible that our study has a higher estimate because one-third of the cohorts recruited based on parental history of asthma or allergies.^32^ Although overall rates of AD did not differ by cohort type, cohort type was associated with AD phenotype. We found that children recruited from high-risk cohorts were more likely to have early AD but less likely to have late or persistent AD.

Early life dog exposure and indoor cat exposure was associated with lower risk for persistent AD, while early antibiotic use was associated with increased risk. Of note, we did not collect the reason for antibiotic use; this is similar to previous studies that have demonstrated that dog exposure^33,34,35,36^ and farm animal exposure^37^ are inversely associated with AD. Because it is well established that microbial changes, such as decreased microbial diversity and increased *S. aureus* colonization are associated with AD,^38,39,40,41^ it is possible that these exposures may influence microbiome changes in the children.

Timing of AD expression was associated with the development of other allergic diseases. Phenotypes with early AD were associated with food allergy, while phenotypes with later expression were associated with allergic rhinitis, and AD at any age was associated with asthma. This variation in timing suggests the skin barrier may play an important role in the development of other allergic diseases and that critical time windows for disease development may differ by disease. There are convincing data^42,43^ that allergic sensitization can occur through cutaneous exposure. Our data indicate that AD in infancy is most associated with food allergy, suggesting that impairment of the skin barrier at this time is most important for food allergy. This finding is further supported by our data demonstrating highest food sensitization was associated with phenotypes with early AD. Peanut and egg are important allergens in AD, and are associated with both AD and food allergy among patients with AD. In contrast, disruption of the skin barrier later in childhood was associated with allergic rhinitis and asthma diagnosis, suggesting that the skin has a potential role in sensitization to aeroallergens.^44^ Finally, persistent AD throughout childhood was associated with all diagnoses (food allergy, asthma, and allergic rhinitis), and may suggest that persistent inflammation and immune activation at the skin barrier leads to most marked Th2 skewing and disease development.

Racially and ethnically minoritized children and those born in more recent high-risk cohorts were at higher risk for early and/or persistent AD. Children from newer cohorts being at higher risk suggests that prevalence may be continuing to increase. Analysis from the ISAAC studies suggested that AD may be increasing but perhaps leveling off in more endemic areas.^10^ Our data suggest that AD may still be increasing. Black children were at higher risk—even after accounting for cohort type, decade of birth, and child sex in multivariable models—illustrating health disparities in AD,^45^ which have been consistently observed in other allergic diseases and asthma.^46,47,48,49^ Because race is a social construct, future work is required, to identify the environmental factors and systems that contribute to these disparities.^50,51,52^ Type of cohort (high risk vs general population) and parental atopy also were associated with AD phenotype, suggesting that in addition to environmental exposures, genetic difference may also influence AD phenotype and the subject of ongoing investigation.

### Strengths and Limitations

Strengths of this work include examination of a large, diverse, nation-wide cohort, and longitudinal dataset, allowing for a more detailed and nuanced examination of phenotypes and relationships to exposures. These data also highlight potential areas for further investigation, including health disparities in AD, genetic influences given the difference in risk with parental atopy, areas of investigation regarding cause of the atopic march (such as timing of allergic sensitization), as well as demonstrate targets and populations for specific interventions.

This study also has limitations. Data harmonization across many cohorts, where data may have been collected in different ways and at different time points is a limitation of the study. Regarding risk factors, we did not collect the reason for antibiotic use. We focused on antibiotic use in the first year of life, and it is possible the antibiotics may have been prescribed for AD or other reasons (such as otitis media or other common reasons). Additionally, we did not assess AD severity in this study, and this is important to include in future work to assess both risk factors and further atopic disease expression.

## Conclusions

In this large US cohort study, we identified 5 AD phenotypes. These phenotypes differed in risk factors including personal characteristics and modifiable early life exposures such as antibiotic use and pet exposure. Identifying AD phenotypes may inform targeted prevention strategies using precision medicine approaches to reduce AD and potentially associated comorbid conditions.

## Notes

Supplement 1.
Nonauthor Collaborators

Supplement 2. **eMethods.** Definitions **eFigure 1.** Distribution of AD Responses by Cohort **eFigure 2.** AD Prevalence by Cohort Type **eTable 1.** Additional Characteristics of CREW Children Included in Analysis Subset (N=5,314) **eTable 2.** AD Phenotypes LLCA Model Results and Fit Statistics **eFigure 3.** Distribution of Maximum Posterior Probabilities by AD Phenotype **eTable 3.** AD Phenotype Sample Sizes and Frequencies by Cohort **eTable 4.** Association Between Additional Modifiable Risk Factors and AD Phenotype (N=5,314) **eFigure 4.** Conditional Inference Tree for Association Between Household, Family, and Child Characteristics and AD Phenotype **eTable 5.** Association Between AD Phenotype and Allergic, Wheeze and Asthma Outcomes (N=5,314)

Supplement 3.
Data Sharing Statement
