# Supplementary material for: Phenotypes of Atopic Dermatitis and Development of Allergic Diseases
Source: JAMA Netw Open. 2025 Jun 12;8(6):e2515094. doi: 10.1001/jamanetworkopen.2025.15094 (PMC12163678; doi:10.1001/jamanetworkopen.2025.15094)
Supplement: Supplement 1. — Nonauthor Collaborators [file jamanetwopen-e2515094-s001.pdf]

Supplemental Online Content: Nonauthor Collaborators
*First name, last name, and suffix (if applicable) are required and will appear in PubMed.

*Group Name: Children's Respiratory and Environmental Workgroup							
***First Name and Middle Initial(s)**	***Last Name**	***Suffix (eg, Jr, III)**	Academic Degrees	Institution	Location (city, state/province, country)	Role or Contribution, eg, chair, principal investigator	Group (if more than 1 Group listed in the byline) and/or Subgroup (eg, Steering Committee)
Leonard B.	Bacharier		MD	Monroe Carell Jr Children's Hospital at Vanderbilt	Nashville, TN	Investigator	URECA
Paloma I.	Beamer		PhD	Asthma and Airway Disease Research Center, University of Arizona	Tucson, AZ	Coinvestigator	IIS
Dean	Billheimer		PhD	University of Arizona	Tucson, AZ	Biostatistician	IIS
Alex	Binder			University of Wisconsin-Madison	Madison, WI	Data Scientist	Informatics Team
Gordon	Bloomberg			Washington University School of Medicine	St Louis, MO	Coinvestigator	URECA
Yury A.	Bochkov		PhD	University of Wisconsin-Madison	Madison, WI	Coinvestigator	COAST, WISC
Steven M.	Brunwasser		PhD	Vanderbilt University Medical Center	Nashville, TN	Coinvestigator	INSPIRE
Tara F.	Carr		MD	University of Arizona	Tucson, AZ	Investigator	IIS
Teresa M.	Chipps		BS	Vanderbilt University Medical Center	Nashville, TN	Program Manager	INSPIRE
Gina	Crisafi			University of Wisconsin-Madison	Madison, WI	Program Manager	Administrative Center
Suman R.	Das		PhD	Division of Infectious Disease, Vanderbilt Technologies for Advanced Genomics	Nashville, TN	Coinvestigator	INSPIRE
Brent	Davidson			Henry Ford Health	Detroit, MI	Coinvestigator, OB	CAS
William D.	Dupont		PhD	Vanderbilt University Medical Center	Nashville, TN	Coinvestigator, Biostatistician	INSPIRE
Abby	Engelhart			Vanderbilt University Medical Center	Nashville, TN	Program Manager	INSPIRE
Samantha	Fye			University of Wisconsin-Madison	Madison, WI	Coinvestigator	WISC
Ronald E.	Gangnon			University of Wisconsin-Madison	Madison, WI	Biostatistician, Data Manager	COAST, WISC

Supplemental Online Content: Nonauthor Collaborators
*First name, last name, and suffix (if applicable) are required and will appear in PubMed.

***First Name and Middle Initial(s)**	***Last Name**	***Suffix (eg, Jr, III)**	Academic Degrees	Institution	Location (city, state/province, country)	Role or Contribution, eg, chair, principal investigator	Group (if more than 1 Group listed in the byline) and/or Subgroup (eg, Steering Committee)
Tebeb	Gebretsadik		MPH	Vanderbilt University Medical Center	Nashville, TN	Coinvestigator, Biostatistician	INSPIRE
Brian	Hallmark		PhD	University of Arizona	Tucson, AZ	Biostatistician	IIS
Marilyn	Halonen		PhD	University of Arizona	Tucson, AZ	Investigator	IIS
Julie	Herbstman			Columbia Center for Children's Environmental Health, Columbia University Medical Center	New York, NY	Principal Investigator	CCCEH
Molly	Johnson			Rho, Inc., Federal Research Operations, Durham, NC	Durham, NC	Senior Biostatistician	URECA
Meyer	Kattan			Columbia University Medical Center	New York, NY	Principal Investigator	URECA
Matthew C.	Keifer			Marshfield Clinic Research Institute	Marshfield, WI	Principal Investigator, retired	WISC
Carin	Lamm		MD	Columbia University Medical Center	New York, NY	Investigator	URECA
Kristine	Lee			University of Wisconsin-Madison	Madison, WI	Biostatistician	WISC
Robert F.	Lemanske	Jr.	MD	University of Wisconsin-Madison	Madison, WI	Principal Investigator	COAST
Grace K.	LeMasters			Cincinnati’s Children Hospital Medical Center	Cincinnati, OH	Investigator	CCAAPS
Stephanie	Lovinsky-Desir			Columbia University Medical Center	New York, NY	Investigator	URECA
Jomol	Matthew			University of Wisconsin-Madison	Madison, WI	Informatics Lead	Informatics Team
Christopher G.	McKennan		PhD	Vanderbilt University Medical Center	Pittsburgh, PA	Biostatistician	INSPIRE
Wayne J.	Morgan		MD	University of Arizona	Tucson, AZ	Investigator	IIS
Mariné	Nalbandyan			University of Wisconsin-Madison	Madison, WI	Data Scientist	Informatics Team
George T.	O’Connor			Boston University School of Medicine	Boston, MA	Principal Investigator	URECA
Irene	Ong			University of Wisconsin-Madison	Madison, WI	Coinvestigator, Biostatistician	WISC

Supplemental Online Content: Nonauthor Collaborators
*First name, last name, and suffix (if applicable) are required and will appear in PubMed.

***First Name and Middle Initial(s)**	***Last Name**	***Suffix (eg, Jr, III)**	Academic Degrees	Institution	Location (city, state/province, country)	Role or Contribution, eg, chair, principal investigator	Group (if more than 1 Group listed in the byline) and/or Subgroup (eg, Steering Committee)
R. Stokes	Peebles		MD, MS	Vanderbilt University Medical Center	Nashville, TN	Coinvestigator	INSPIRE
Frederica	Perera		DrPH, PhD	Columbia Center for Children's Environmental Health, Columbia University Medical Center	New York, NY	Investigator	CCCEH
Matthew	Perzanowski		PhD	Columbia Center for Children's Environmental Health, Columbia University Medical Center	New York, NY	Investigator	CCCEH
Chris M	Reyes		MS	University of Wisconsin-Madison	Madison, WI	Publications Director	Administrative Center
Christian	Rosas-Salazar		MD, MPH	Vanderbilt University Medical Center	Nashville, TN	Coinvestigator	INSPIRE
Patrick H.	Ryan		PhD	Department of Pediatrics and College of Medicine, University of Cincinnati	Cincinnati, OH	Principal Investigator	CCAAPS
Hugh	Sampson			Icahn School of Medicine at Mount Sinai	New York, NY	Investigator	URECA
Megan T.	Sandel			Boston University School of Medicine	Boston, MA	Investigator	URECA
Ruchika	Sangani			Boston University School of Medicine	Boston, MA	Investigator	URECA
Meghan H.	Shilts		MS, MHS	Division of Infectious Disease, Vanderbilt Technologies for Advanced Genomics	Nashville, TN	Senior Research Specialist	INSPIRE
Akihiro	Shiroshita			Vanderbilt University Medical Center	Nashville, TN	Coinvestigator (PhD student)	INSPIRE
Brittney M.	Snyder		PhD	Vanderbilt University Medical Center	Nashville, TN	Coinvestigator	INSPIRE
Ronald	Sorkness			University of Wisconsin-Madison	Madison, WI	Coinvestigator	COAST
Rhoda	Sperling			Icahn School of Medicine at Mount Sinai	New York, NY	Investigator	URECA

Supplemental Online Content: Nonauthor Collaborators
*First name, last name, and suffix (if applicable) are required and will appear in PubMed.

***First Name and Middle Initial(s)**	***Last Name**	***Suffix (eg, Jr, III)**	Academic Degrees	Institution	Location (city, state/province, country)	Role or Contribution, eg, chair, principal investigator	Group (if more than 1 Group listed in the byline) and/or Subgroup (eg, Steering Committee)
Jeff	Stokes			Washington University School of Medicine	St Louis, MO	Coinvestigator	URECA
Zhengzheng	Tang		PhD	Department of Biostatistics and Medical Informatics, University of Wisconsin	Madison, WI	Data Analyst	WISC
Kedir	Turi		PhD	Vanderbilt University Medical Center	Nashville, TN	Coinvestigator	INSPIRE
Jeffrey J.	VanWormer			Marshfield Clinic Research Institute	Marshfield, WI	Co-Principal Investigator	WISC
Cynthia M.	Visness		PhD, MPH	Rho, Inc., Federal Research Operations, Durham, NC	Durham, NC	Senior Research Scientist	URECA
Robert A.	Wood		MD	Johns Hopkins University School of Medicine	Baltimore, MD	Principal Investigator	URECA
Rosalind J.	Wright			Icahn School of Medicine at Mount Sinai	New York, NY	Investigator	URECA
Melissa	Yaeger			University of Wisconsin-Madison	Madison, WI	Program Manager	Administrative Center
Edward M.	Zoratti		MD	Henry Ford Health	Detroit, MI	Principal Investigator	CAS
