# Supplementary material for: Phenotypes of Atopic Dermatitis and Development of Allergic Diseases
Source: JAMA Netw Open. 2025 Jun 12;8(6):e2515094. doi: 10.1001/jamanetworkopen.2025.15094 (PMC12163678; doi:10.1001/jamanetworkopen.2025.15094)
Supplement: Supplement 2. — eMethods. Definitions eFigure 1. Distribution of AD Responses by Cohort eFigure 2. AD Prevalence by Cohort Type eTable 1. Additional Characteristics of CREW Children Included in Analysis Subset (N=5,314) eTable 2. AD Phenotypes LLCA Model Results and Fit Statistics eFigure 3. Distribution of Maximum Posterior Probabilities by AD Phenotype eTable 3. AD Phenotype Sample Sizes and Frequencies by Cohort eTable 4. Association Between Additional Modifiable Risk Factors and AD Phenotype (N=5,314) eFigure 4. Conditional Inference Tree for Association Between Household, Family, and Child Characteristics and AD Phenotype eTable 5. Association Between AD Phenotype and Allergic, Wheeze and Asthma Outcomes (N=5,314) [file jamanetwopen-e2515094-s002.pdf]

Supplemental Online Content

Sitarik AR, Eapen AA, Biagini JM, et al. Phenotypes of atopic dermatitis and development of allergic diseases. *JAMA Netw. Open*. 2025;8(6):e2515094. doi:10.1001/jamanetworkopen.2025.15094

eMethods. Definitions
eFigure 1. Distribution of AD Responses by Cohort
eFigure 2. AD Prevalence by Cohort Type
eTable 1. Additional Characteristics of CREW Children Included in Analysis Subset (N=5,314) eTable 2: AD Phenotypes LLCA Model Results and Fit Statistics
eFigure 3. Distribution of Maximum Posterior Probabilities by AD Phenotype
eTable 3. AD Phenotype Sample Sizes and Frequencies by Cohort
eTable 4. Association Between Additional Modifiable Risk Factors and AD Phenotype (N=5,314)
eFigure 4. Conditional Inference Tree for Association Between Household, Family, and Child Characteristics and AD Phenotype
eTable 5. Association Between AD Phenotype and Allergic, Wheeze and Asthma Outcomes (N=5,314)

This supplemental material has been provided by the authors to give readers additional information about their work.

© 2025 Sitarik AR et al. *JAMA Network Open*.

e Methods. Definitions

Biomarkers

Aeroallergen sensitization
Aeroallergen skin prick testing (SPT) and specific IgE (sIgE) were assessed. SPT was considered positive if greater than or equal to 3 mm above the negative control (unless negative control results were not available, in which case 2:3 mm alone was considered positive). One exception was the CAS study defined a positive test as wheal 2: 4 mm, flare 2: 10mm, and control wheal = 0 mm.

For sIgE, positivity was defined as 2:0.35 kU/L. Some centers utilized allergen mixes (e.g., mold mix, grass mix) while others utilized single specific allergens. To prevent overrepresentation, we filtered the data into aeroallergen categories and counted as positive if at least one allergen in the category was positive. We further categorized the allergens into perennial (cockroach, mouse/rat, house dust, dust mite, cat, dog) and seasonal (grass, weeds, tree, *Alternaria*).

We calculated scores for seasonal, perennial and total respiratory allergies by dividing the number of positive allergen tests from each category by the total number of allergen tests. As testing was performed at different ages across cohorts, test results were grouped as follows: 24-36 months, 60-84 months, and 120 months. For sIgEs, 12, 18-24, 36, 60, 72-84, and 108-156 months were used. If a child was tested multiple times within a specific age group as above, the time point with the most positive tests within that age group was used.

Food sensitization
Specific IgE for egg white, cow’s milk, and peanut was considered positive if 2: 0.35 for all cohorts except EHAAS, where >0.35 was considered positive and 0.35 was considered negative, as previously defined by the EHAAS investigators. Data were assessed at the following ages: 12 months, 24 months, 36-48 months, and 60-72 months.

Peripheral Eosinophils
There were 7 cohorts that collected samples for total peripheral blood eosinophils and percent eosinophils (TCRS, IIS, COAST, URECA, EHAAS, WHEALS and CAS). Only percentages but not counts were reported for TCRC and IIS, and were converted to total eosinophil counts based on total white blood cell counts. Because peripheral eosinophilia tends to remain relatively stable during the first decade of life and years of life,[1] data for eosinophil counts were used as close to the age of 5-6 years for available cohorts. However, WHEALS had eosinophil data only at age 10 while CAS had eosinophil data only at age 18. Counts were reported as cells/mm^3.

Total IgE
We assessed the trajectory of total IgE from all available ages (cord blood to 18 years). Latent class growth analysis (LCGA) was applied to the data, where three trajectories were selected, which identified low, medium, and high total IgE trajectory classes, which were used for downstream analyses.

Antibiotic use
Antibiotic use during this time period was assessed in eight cohorts (MAAP, IIS, URECA, CCAAPS, INSPIRE, EHAAS, WHEALS, CAS).

Covariates

Birth Order
Birth order was collected by parental report or chart review.

Breast feeding
A child was reported as being breastfed (ever) if mother reported breast feeding at any time.

Delivery Mode
Delivery mode (vaginal or cesarean section) was determined by parental report at the first visit after the child was born, or from the medical record.

© 2025 Sitarik AR et al. *JAMA Network Open*.

Gestational Age
Gestational age (in weeks) at birth was collected.

Pet Ownership
All cohorts reported information on presence of cats and dogs in the home during the first year of life, though only some provided indoor/outdoor information. Animals were considered "indoor" if they spent any time indoors. The harmonized age intervals were during the first year of life and 1 to <5 years of age.

Smoke exposure
Prenatal smoke exposure was defined as mother reported smoking during pregnancy. Infancy (birth to age 12 months) and childhood (1 to <5 years old) smoke exposure was defined as if the mother or any other person in the household reported smoking during those time periods.

Race
Child's race was determined by parental report, as previously described [2]. Harmonized racial categories included American Indian or Alaska Native, Asian, Black/African American, Native Hawaiian/Pacific Islander, White, and Other/Multiracial. Due to small sample sizes, American Indian or Alaska Native, Asian, and Native Hawaiian/Pacific Islander were collapsed into the Other/Multiracial category for analysis purposes.

Model Selection

The best-fitting LLCA model was selected by considering a variety of model fit statistics, classification accuracy, and clinical interpretability of the class solutions. The primary LLCA was based on the dataset including missing values (in which missing at random is assumed). However, as a sensitivity analysis, AD at each year of age was multiply imputed to examine the stability in the best-fitting number of classes. Multilevel data imputation was performed using the "mice" package in R [3], which accounts for within-subject correlation in longitudinal AD status. A total of 5 imputed datasets were calculated, using a variety of maternal, child, and household factors to impute AD; LLCA was then fit on each of the 5 imputed datasets and fit statistics were averaged.

After the best-fitting number of classes was determined, the association between household, family, and child characteristics and AD phenotype was examined using a 3-step approach for latent class modeling with covariates, using the R3STEP setting in Mplus [4]. This approach uses class assignment uncertainty in model estimation to calculate odds ratios (ORs) and corresponding 95% confidence intervals (CIs) based on multinomial logistic regression models, using AD phenotype as the outcome. All models were adjusted for cohort type (high risk vs. general risk), decade of birth (2000s-2010s vs. 1980s-1990s), child sex (male vs. female), and child race (Black vs. White vs. Other/Multiracial), which were considered a minimal set of essential variables that explained a large proportion of variation between CREW subjects. In order to determine what factors had the strongest associations with AD phenotype and explore potential interactions, conditional inference trees were built using the "partykit" R package [5]. Briefly, a permutation-based global null hypothesis of independence between any of the covariates and AD phenotype was tested; the decision tree stops splitting when the null cannot be rejected [6]. All default parameters were used (including Bonferroni-adjusted p-values), with the exception that the minimum terminal node size was set to 50 rather than the default of 7.

References

1. Anderson HM, Lemanske RF, Jr., Arron JR, et al. Relationships among aeroallergen sensitization, peripheral blood eosinophils, and periostin in pediatric asthma development. *J Allergy Clin Immunol.* 2017;139(3):790-796.
2. Johnson CC, Havstad SL, Ownby DR, et al. Pediatric asthma incidence rates in the United States from 1980 to 2017. *J Allergy Clin Immunol.* 2021;148(5):1270-1280.
3. van Buuren S, Groothuis-Oudshoorn K. mice: Multivariate Imputation by Chained Equations in R. *Journal of Statistical Software.* 2011;45(3):1 - 67.
4. Asparouhov T, Muthén B. Auxiliary Variables in Mixture Modeling: Three-Step Approaches Using Mplus. *Structural Equation Modeling: A Multidisciplinary Journal.* 2014;21(3):329-341.

© 2025 Sitarik AR et al. *JAMA Network Open*.

5. Hothorn T, Zeileis A. Partykit: a modular toolkit for recursive partytioning in R. *J Mach Learn Res.* 2015;16(1):3905–3909.
6. Hothorn T, Hornik K, Zeileis A. Unbiased Recursive Partitioning: A Conditional Inference Framework. *Journal of Computational and Graphical Statistics*. 2006;15(3):651-674.

© 2025 Sitarik AR et al. *JAMA Network Open*.

eFigure 1: Distribution of AD Responses by Cohort.

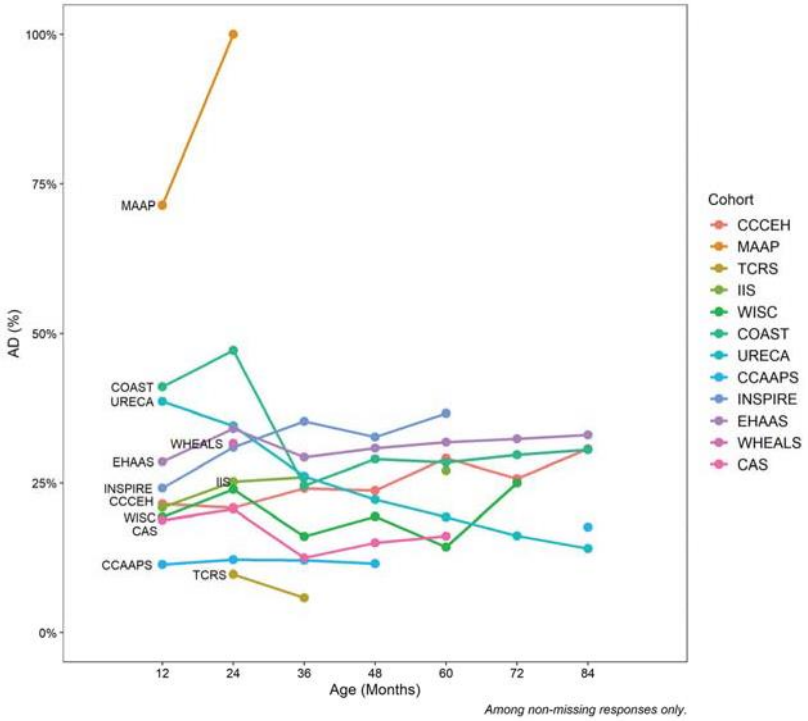

MAAP, TCRS, WHEALS were not included as fewer than 3 time points were assessed.

© 2025 Sitarik AR et al. *JAMA Network Open*.

eFigure 2: AD Prevalence by Cohort Type

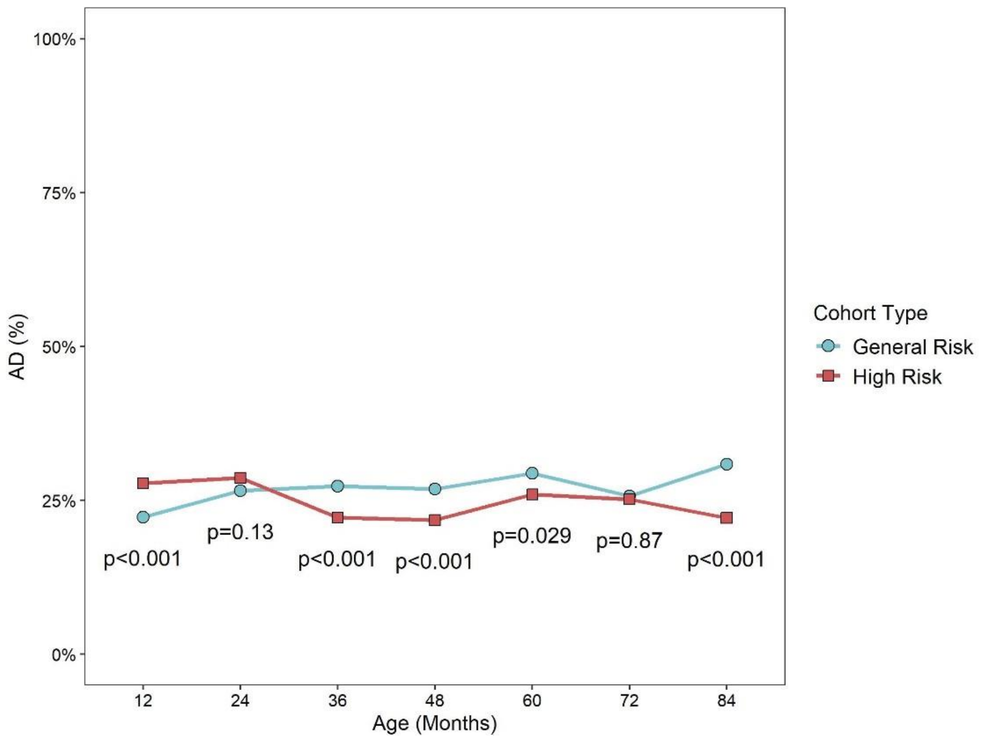

© 2025 Sitarik AR et al. *JAMA Network Open*.

eTable 1: Additional characteristics of CREW children included in analysis subset (N=5,314)

Characteristic	n (%)
**Delivery Mode**	
Vaginal	2623 (49.4%)
C-section	982 (18.5%)
Missing	1709 (32.2%)
**First Born Child**	
No	996 (18.7%)
Yes	945 (17.8%)
Missing	3373 (63.5%)
**Ever Breastfed**	
No	1073 (20.2%)
Yes	3629 (68.3%)
Missing	612 (11.5%)
**Antibiotic Use in the First Year of Life**	
No	1444 (27.2%)
Yes	1702 (32.0%)
Missing	2168 (40.8%)
**Daycare in the First Year of Life**	
No	2157 (40.6%)
Yes	1813 (34.1%)
Missing	1344 (25.3%)
**Dogs in the First Year of Life**	
No	3339 (62.8%)
Yes	1898 (35.7%)
Missing	77 (1.4%)
**Indoor Dogs in the First Year of Life**	
No	3657 (68.8%)
Yes	1198 (22.5%)
Missing	459 (8.6%)
**Dogs During Childhood (1 to <5 Years)**	
No	2836 (53.4%)
Yes	2142 (40.3%)
Missing	336 (6.3%)
**Cats in the First Year of Life**	
No	3953 (74.4%)
Yes	1284 (24.2%)
Missing	77 (1.4%)
**Indoor Cats in the First Year of Life**	
No	4385 (82.5%)
Yes	437 (8.2%)
Missing	492 (9.3%)
**Cats During Childhood (1 to <5 Years)**	
No	3556 (66.9%)
Yes	1442 (27.1%)
Missing	316 (5.9%)
**Maternal Smoking During Pregnancy**	
No	4661 (87.7%)
Yes	597 (11.2%)
Missing	56 (1.1%)
**Maternal Smoking During First Year of Life**	

© 2025 Sitarik AR et al. *JAMA Network Open*.

Characteristic	n (%)
No	4021 (75.7%)
Yes	748 (14.1%)
Missing	545 (10.3%)
**Maternal Smoking During Childhood (1 to <5 Years)**	
No	3892 (73.2%)
Yes	1098 (20.7%)
Missing	324 (6.1%)
**Other Household Smokers During Pregnancy**	
No	1295 (24.4%)
Yes	420 (7.9%)
Missing	3599 (67.7%)
**Other Household Smokers During First Year of Life**	
No	3614 (68.0%)
Yes	1399 (26.3%)
Missing	301 (5.7%)
**Other Household Smokers During Childhood (1 to <5 Years)**	
No	3422 (64.4%)
Yes	1639 (30.8%)
Missing	253 (4.8%)
**Maternal Asthma**	
No	2273 (42.8%)
Yes	955 (18.0%)
Missing	2086 (39.3%)
**Paternal Asthma**	
No	2883 (54.3%)
Yes	581 (10.9%)
Missing	1850 (34.8%)
**Sibling Asthma**	
No	2354 (44.3%)
Yes	626 (11.8%)
Missing	2334 (43.9%)
**Gestational Age**	
Mean (SD)	39.2 (1.30)
Missing	93 (1.8%)
**Parental Report of Physician Diagnosed AD at 12 Months**	
No	3880 (73.0%)
Yes	1241 (23.4%)
Missing	193 (3.6%)
**Parental Report of Physician Diagnosed AD at 24 Months**	
No	3582 (67.4%)
Yes	1346 (25.3%)
Missing	386 (7.3%)
**Parental Report of Physician Diagnosed AD at 36 Months**	
No	3676 (69.2%)
Yes	1250 (23.5%)
Missing	388 (7.3%)
**Parental Report of Physician Diagnosed AD at 48 Months**	
No	3353 (63.1%)
Yes	1104 (20.8%)
Missing	857 (16.1%)
**Parental Report of Physician Diagnosed AD at 60 Months**	
No	2916 (54.9%)
Yes	1156 (21.8%)
Missing	1242 (23.4%)

© 2025 Sitarik AR et al. *JAMA Network Open*.

Characteristic	n (%)
**Parental Report of Physician Diagnosed AD at 72 Months**	
No	1239 (23.3%)
Yes	420 (7.9%)
Missing	3655 (68.8%)
**Parental Report of Physician Diagnosed AD at 84 Months**	
No	1701 (32.0%)
Yes	540 (10.2%)
Missing	3073 (57.8%)
**Food Allergy (0-6 Years)**	
No	3458 (65.1%)
Yes	812 (15.3%)
Missing	1044 (19.6%)
**Ever Asthma**	
No	3840 (72.3%)
Yes	1278 (24.0%)
Missing	196 (3.7%)
**Allergic Rhinitis at 2-4 Years**	
No	1407 (26.5%)
Yes	980 (18.4%)
Missing	2927 (55.1%)
**Allergic Rhinitis at 5-7 Years**	
No	2945 (55.4%)
Yes	1136 (21.4%)
Missing	1233 (23.2%)

© 2025 Sitarik AR et al. *JAMA Network Open*.

eTable 2: AD phenotypes LLCA model results and fit statistics

	1 Class	2 Classes	3 Classes	4 Classes	5 Classes	6 Classes	7 Classes
**Unimputed Dataset**							
Log-likelihood	-15615.936	-11942.225	-11718.226	-11551.718	-11524.157	-11510.129	-11499.667
AIC	31245.872	23914.451	23482.452	23165.437	23126.313	23114.257	23109.334
BIC	31291.919	24013.122	23633.748	**23369.358**[a]	23382.859	23423.428	23471.129
SSA-BIC	31269.675	23965.457	23560.662	23270.850	**23258.930**	23274.077	23296.357
LMR p-value	NA	<0.001	<0.001	**<0.001**	0.0741	0.0260	0.089
BLRT p-value	NA	<0.001	<0.001	<0.001	<0.001	<0.001	0.040
Entropy	NA	0.875	0.751	0.775	0.788	0.789	0.732
Error messages?	No	No	No	No	No	No	No
% per class	100%	72%, 28%	17%, 16%, 67%	9%, 15%, 66%, 10%	9%, 5%, 15%, 66%, 4%	4%, 3%, 5%, 15%, 66%, 5%	5%, 3%, 64%, 3%, 3%, 15%, 7%
**Averages Across 5 Imputed Datasets**							
Log-likelihood	-21211.448	-18500.082	-18381.036	-18292.308	-18233.437	-18220.087	-18217.015
AIC	42436.896	37030.164	36808.073	36646.616	36544.874	**36534.173**	36544.031
BIC	42482.943	37128.836	36959.369	36850.537	**36801.420**	36843.344	36905.826
SSA-BIC	42460.699	37081.171	36886.283	36752.029	**36677.491**	36693.993	36731.054
Entropy	NA	0.848	0.812	0.754	0.743	0.753	0.668
Error messages?	No	No	No	No	No	No	No
% per class	100%	25%, 75%	14%, 12%, 74%	7%, 7%, 18%, 68%	7%, 6%, 11%, 68%, 8%	6%, 10%, 3%, 68%, 7%, 6%	1%, 7%, 12%, 6%, 6%, 28%, 40%

[a]*bold values indicate best-fitting class for that metric, if found.*

© 2025 Sitarik AR et al. *JAMA Network Open*.

eFigure 3: Distribution of maximum posterior probabilities by AD phenotype

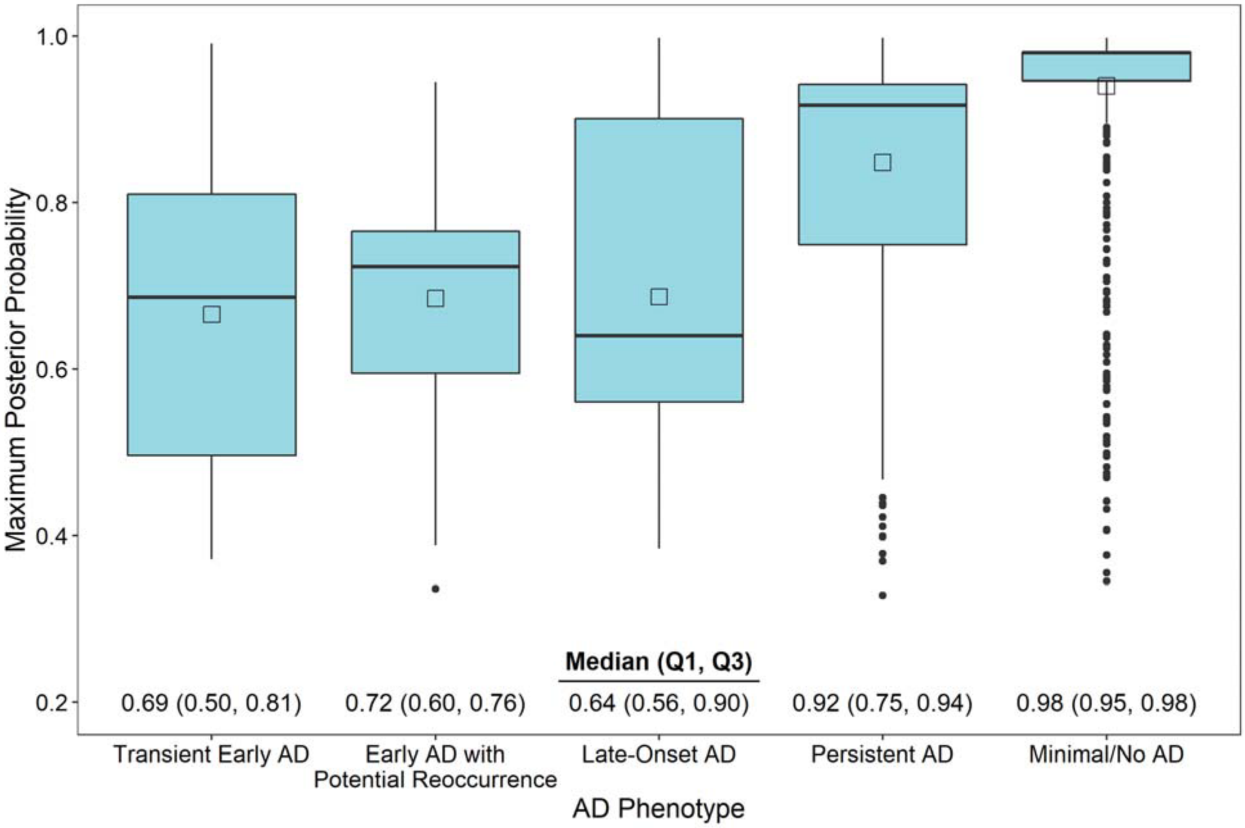

© 2025 Sitarik AR et al. *JAMA Network Open*.

eTable 3: AD phenotype sample sizes and frequencies by cohort

AD Phenotype	CAS	CCAAPS	CCCEH	COAST	EHAAS	IIS	INSPIRE	URECA	WISC
Transient Early AD	25 (3.8%)	18 (2.8%)	14 (2.4%)	19 (6.9%)	27 (5.6%)	9 (2.4%)	77 (4.8%)	69 (13.3%)	3 (1.9%)
Early AD with Potential Reoccurrence	30 (4.5%)	13 (2%)	11 (1.9%)	25 (9.1%)	20 (4.1%)	10 (2.7%)	44 (2.7%)	70 (13.5%)	6 (3.8%)
Late-Onset AD	60 (9%)	41 (6.3%)	56 (9.6%)	25 (9.1%)	46 (9.5%)	31 (8.3%)	182 (11.3%)	32 (6.2%)	5 (3.2%)
Persistent AD	39 (5.9%)	33 (5%)	97 (16.6%)	47 (17%)	109 (22.5%)	69 (18.5%)	367 (22.9%)	42 (8.1%)	18 (11.5%)
Minimal/No AD	509 (76.8%)	549 (83.9%)	408 (69.6%)	160 (58%)	282 (58.3%)	254 (68.1%)	934 (58.2%)	305 (58.9%)	124 (79.5%)

© 2025 Sitarik AR et al. *JAMA Network Open*.

eTable 4: Association between additional modifiable risk factors and AD phenotype (N=5,314)

Characteristic	N	Transient Early AD 261 (4.9%)	Early AD with Potential Reoccurrence 229 (4.3%)	Late-Onset AD 478 (9.0%)	Persistent AD 821 (15.4%)
		OR [95% CI][a]			
**Modifiable Risk Factors**					
Indoor Dogs in the First Year of Life: Yes vs. No	4364	0.65 [0.32, 1.30]	0.28 [0.10, 0.79]	0.87 [0.60, 1.26]	0.87 [0.67, 1.14]
Dogs During Childhood (1 to <5 Years): Yes vs. No	4593	0.79 [0.50, 1.26]	0.56 [0.33, 0.96]	0.92 [0.66, 1.28]	0.79 [0.63, 0.99]
Indoor Cats in the First Year of Life: Yes vs. No	4321	1.33 [0.69, 2.55]	0.76 [0.27, 2.14]	0.44 [0.18, 1.07]	0.31 [0.11, 0.86]
Cats During Childhood (1 to <5 Years): Yes vs. No	4639	0.77 [0.44, 1.33]	0.98 [0.54, 1.77]	0.90 [0.63, 1.28]	0.81 [0.65, 1.02]
Maternal Smoking During Pregnancy: Yes vs. No	4726	0.69 [0.37, 1.29]	0.82 [0.45, 1.50]	0.91 [0.58, 1.44]	0.63 [0.46, 0.88]
Maternal Smoking During Childhood (1 to <5 Years): Yes vs. No	4520	0.69 [0.42, 1.14]	1.14 [0.71, 1.83]	0.80 [0.54, 1.18]	0.73 [0.57, 0.93]
Other Household Smokers During Pregnancy: Yes vs. No	1235	1.08 [0.35, 3.36]	1.98 [0.99, 3.99]	1.11 [0.34, 3.66]	1.01 [0.58, 1.76]
Other Household Smokers During Childhood (1 to <5 Years): Yes vs. No	4593	0.97 [0.61, 1.56]	0.99 [0.63, 1.57]	0.92 [0.66, 1.29]	0.76 [0.59, 0.98]

[a]*Odds ratios and 95% CIs were estimated using the multinomial logistic regression for each covariate with the 3-step procedure described by Asparouhov and Muthén 2014 [4]. All odds ratios are relative to the Minimal/No AD class, which was comprised of 3525 (66.3%) children, and are adjusted for cohort type, decade of birth, child sex, and child race. Significant ORs>1 are highlighted in red, while significant ORs<1 are highlighted in blue.*

© 2025 Sitarik AR et al. *JAMA Network Open*.

eFigure 4: Conditional inference tree for association between household, family, and child characteristics and AD phenotype

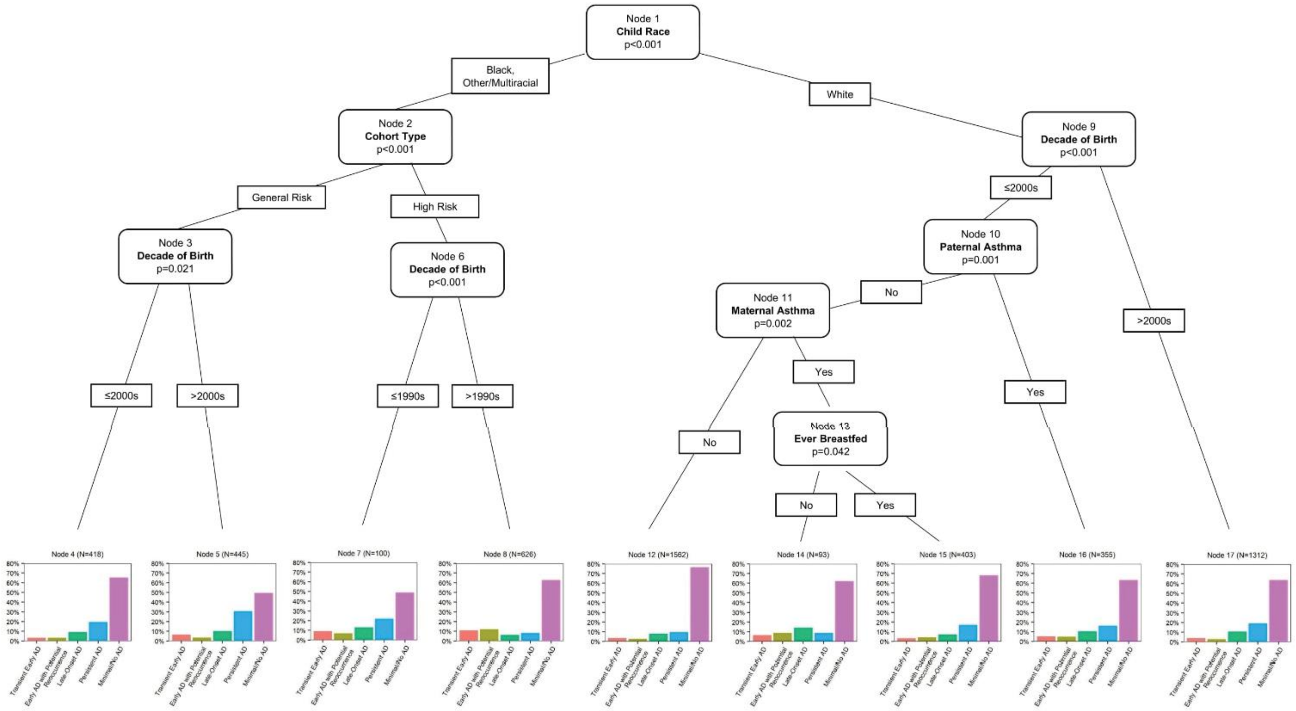

© 2025 Sitarik AR et al. *JAMA Network Open*.

eTable 5: Association between AD phenotype and allergic, wheeze and asthma outcomes (N=5,314)
Boxes shaded in red represent variables with significantly increased risk. Significant ORs>1 (risk factors) are highlighted in red, while significant ORs<1 (protective factors) are highlighted in blue.

Outcome	N	Transient Early AD 261 (4.9%)	Early AD with Potential Reoccurrence 229 (4.3%)	Late-Onset AD 478 (9.0%)	Persistent AD 821 (15.4%)	Minimal/ No AD 3525 (66.3%)
		OR [95% CI][a]				
Food Allergy (0-6 Years)	4270	2.15 [1.48, 3.08]	2.43 [1.66, 3.50]	1.21 [0.87, 1.65]	2.26 [1.84, 2.78]	1.0 [reference]
Egg sIgE Sensitization						
12 Months	1998	2.02 [1.14, 3.43]	1.53 [0.81, 2.73]	1.31 [0.75, 2.19]	5.11 [3.76, 6.96]	1.0 [reference]
24 Months	1172	1.61 [0.86, 2.85]	1.72 [0.88, 3.19]	0.67 [0.29, 1.37]	2.35 [1.54, 3.55]	1.0 [reference]
36-48 Months	1041	2.06 [1.15, 3.61]	1.48 [0.77, 2.72]	1.02 [0.48, 1.96]	2.40 [1.54, 3.71]	1.0 [reference]
60-72 Months	942	2.96 [1.63, 5.26]	1.18 [0.53, 2.39]	1.23 [0.56, 2.45]	2.95 [1.82, 4.75]	1.0 [reference]
Milk sIgE Sensitization						
12 Months	1998	1.26 [0.67, 2.24]	0.73 [0.33, 1.43]	0.75 [0.37, 1.40]	2.16 [1.49, 3.10]	1.0 [reference]
24 Months	1002	1.57 [0.87, 2.78]	1.15 [0.58, 2.15]	0.96 [0.44, 1.91]	2.07 [1.25, 3.39]	1.0 [reference]
36-48 Months	972	1.59 [0.92, 2.71]	1.23 [0.68, 2.16]	0.78 [0.36, 1.53]	1.87 [1.13, 3.07]	1.0 [reference]
60-72 Months	715	1.53 [0.81, 2.80]	0.90 [0.41, 1.83]	0.63 [0.22, 1.54]	2.70 [1.41, 5.10]	1.0 [reference]
Peanut sIgE Sensitization						
12 Months	1999	2.36 [1.19, 4.41]	1.59 [0.72, 3.22]	1.02 [0.43, 2.12]	5.35 [3.66, 7.86]	1.0 [reference]
24 Months	1004	3.31 [1.47, 7.05]	1.05 [0.30, 2.87]	1.19 [0.32, 3.34]	5.45 [2.98, 9.93]	1.0 [reference]
36-48 Months	974	2.71 [1.25, 5.52]	1.98 [0.83, 4.31]	0.81 [0.18, 2.46]	3.99 [2.11, 7.41]	1.0 [reference]
60-72 Months	942	1.90 [0.92, 3.70]	2.05 [0.98, 4.05]	1.69 [0.72, 3.59]	2.98 [1.68, 5.21]	1.0 [reference]
Perennial Aeroallergen sIgE Sensitization						
12 Months	1288	2.03 [0.68, 5.03]	1.40 [0.42, 3.68]	0.88 [0.25, 2.30]	5.38 [3.33, 8.76]	1.0 [reference]
18-24 Months	1591	1.78 [0.80, 3.64]	3.42 [1.69, 6.63]	2.40 [1.17, 4.63]	3.68 [2.25, 6.00]	1.0 [reference]
36 Months	1508	1.34 [0.70, 2.43]	3.34 [1.94, 5.67]	1.50 [0.77, 2.75]	4.18 [2.77, 6.30]	1.0 [reference]
60 Months	1438	1.54 [0.87, 2.65]	2.44 [1.40, 4.20]	1.40 [0.77, 2.46]	3.12 [2.10, 4.63]	1.0 [reference]
72-84 Months	1069	1.58 [0.92, 2.69]	2.31 [1.34, 3.98]	1.26 [0.71, 2.18]	2.86 [1.85, 4.44]	1.0 [reference]
108-156 Months	1078	1.59 [0.94, 2.70]	1.49 [0.84, 2.66]	1.10 [0.67, 1.81]	2.06 [1.40, 3.06]	1.0 [reference]

© 2025 Sitarik AR et al. *JAMA Network Open*.

Outcome	N	Transient Early AD 261 (4.9%)	Early AD with Potential Reoccurrence 229 (4.3%)	Late-Onset AD 478 (9.0%)	Persistent AD 821 (15.4%)	Minimal/ No AD 3525 (66.3%)
		OR [95% CI][a]				
Seasonal Aeroallergen sIgE Sensitization						
12 Months	1290	NA[b]	NA	NA	2.90 [0.67, 12.43]	1.0 [reference]
18-24 Months	1045	NA	1.23 [0.26, 3.93]	0.60 [0.07, 2.39]	2.68 [1.32, 5.24]	1.0 [reference]
36 Months	985	0.74 [0.24, 1.84]	1.13 [0.43, 2.56]	0.72 [0.22, 1.83]	2.32 [1.36, 3.89]	1.0 [reference]
60 Months	954	1.12 [0.54, 2.18]	1.75 [0.89, 3.30]	1.12 [0.56, 2.12]	2.46 [1.57, 3.82]	1.0 [reference]
72-84 Months	1060	0.95 [0.46, 1.82]	1.62 [0.86, 2.92]	1.50 [0.79, 2.74]	3.46 [2.19, 5.47]	1.0 [reference]
108-156 Months	904	1.20 [0.65, 2.17]	1.26 [0.64, 2.37]	0.92 [0.51, 1.64]	1.97 [1.29, 3.00]	1.0 [reference]
Perennial Aeroallergen SPT Sensitization						
24-36 Months	2075	1.67 [1.07, 2.57]	1.68 [1.01, 2.77]	1.71 [1.18, 2.45]	2.11 [1.61, 2.77]	1.0 [reference]
60-84 Months	1813	2.05 [1.34, 3.15]	1.66 [1.03, 2.65]	1.07 [0.69, 1.64]	3.12 [2.24, 4.35]	1.0 [reference]
120 Months	374	NA	NA	NA	NA	1.0 [reference]
Seasonal Aeroallergen SPT Sensitization						
24-36 Months	2073	0.55 [0.31, 0.94]	0.93 [0.52, 1.59]	1.11 [0.74, 1.64]	2.33 [1.76, 3.09]	1.0 [reference]
60-84 Months	1813	1.31 [0.84, 2.01]	2.18 [1.37, 3.45]	1.26 [0.83, 1.88]	3.07 [2.23, 4.25]	1.0 [reference]
120 Months	374	NA	NA	NA	NA	1.0 [reference]
Ever asthma	5118	1.71 [1.22, 2.37]	1.60 [1.13, 2.26]	1.43 [1.08, 1.88]	2.31 [1.91, 2.79]	1.0 [reference]
Wheezing						
Year 1	3089	1.28 [0.88, 1.86]	1.24 [0.84, 1.83]	1.22 [0.88, 1.67]	1.38 [1.09, 1.75]	1.0 [reference]
Year 2	2856	1.90 [1.29, 2.79]	1.51 [0.99, 2.27]	1.36 [0.94, 1.94]	1.98 [1.53, 2.56]	1.0 [reference]
Year 3	2747	2.20 [1.47, 3.26]	1.34 [0.84, 2.10]	1.39 [0.93, 2.03]	2.42 [1.84, 3.16]	1.0 [reference]
Year 4	2351	1.96 [1.26, 2.99]	1.43 [0.86, 2.30]	1.75 [1.14, 2.63]	2.40 [1.76, 3.26]	1.0 [reference]
Year 5	2164	1.35 [0.82, 2.17]	1.33 [0.79, 2.16]	1.55 [0.99, 2.37]	2.77 [2.06, 3.71]	1.0 [reference]
Allergic Rhinitis						
2-4 Years	2387	0.75 [0.48, 1.15]	0.95 [0.60, 1.49]	1.15 [0.79, 1.67]	1.46 [1.11, 1.93]	1.0 [reference]
5-7 Years	4081	0.95 [0.65, 1.37]	1.31 [0.88, 1.92]	1.84 [1.38, 2.43]	2.02 [1.64, 2.48]	1.0 [reference]
Total IgE Trajectory Class	3328					

© 2025 Sitarik AR et al. *JAMA Network Open*.

Outcome	N	Transient Early AD 261 (4.9%)	Early AD with Potential Reoccurrence 229 (4.3%)	Late-Onset AD 478 (9.0%)	Persistent AD 821 (15.4%)	Minimal/ No AD 3525 (66.3%)
		OR [95% CI][a]				
Low		1.0 [reference]	1.0 [reference]	1.0 [reference]	1.0 [reference]	1.0 [reference]
Medium		0.79 [0.51, 1.24]	0.89 [0.55, 1.44]	0.87 [0.61, 1.23]	2.09 [1.56, 2.81]	1.0 [reference]
High		1.43 [0.80, 2.57]	1.31 [0.69, 2.50]	1.27 [0.76, 2.14]	3.71 [2.52, 5.45]	1.0 [reference]
Increased blood eosinophils[c]	1097	43.9% [2.2%, 102.6%]	42.7% [-0.9%, 105.4%]	12.5% [-21.4%, 61.0%]	62.9% [23.9%, 114.1%]	0.0 [reference]

[a]*Unless noted otherwise, estimates are odds ratios and 95% CIs calculated using logistic regression for 2 categories or multinomial logistic regression for >2 categories), with maximum posterior probability used as subject weight. All models are adjusted for cohort type, decade of birth, child sex, and child race. Significant ORs>1 are highlighted in red, while significant ORs<1 are highlighted in blue.*

[b]*Unstable estimate or model did not converge.*

[c]*Percent increase in eosinophil count (cells/mm^3) relative to Minimal/No AD.*

© 2025 Sitarik AR et al. *JAMA Network Open*.
