# Supplementary material for: Phenotypes of Atopic Dermatitis and Development of Allergic Diseases
Source: JAMA Netw Open. 2025 Jun 12;8(6):e2515094. doi: 10.1001/jamanetworkopen.2025.15094 (PMC12163678; doi:10.1001/jamanetworkopen.2025.15094)
Supplement: Supplement 3. — Data Sharing Statement [file jamanetwopen-e2515094-s003.pdf]

Data Sharing Statement

Sitarik. Phenotypes of Atopic Dermatitis and Development of Allergic Diseases. *JAMA Netw Open*. Published June 12, 2025. doi:10.1001/jamanetworkopen.2025.15094

Data
Data available: Yes
Data types: Deidentified participant data
How to access data: Data will be shared on ImmPORT, but is restricted to studies of allergy and asthma.
When available: With publication

Supporting Documents
Document types: None

Additional Information
Who can access the data: Data can only be used for studies on asthma and immunologic diseases.
Types of analyses: Research
Mechanisms of data availability: ImmPORT
Any additional restrictions: Data can only be used for studies on asthma and immunologic diseases.
